# Investigation of the Gut Microbiome in Treatment-Resistant Schizophrenia Compared to Healthy Controls: A Cross-Sectional Pilot Study: Étude du microbiome intestinal chez les patients atteints de schizophrénie résistante au traitement par rapport aux témoins sains : étude pilote transversale

**DOI:** 10.1177/07067437261487782

**Published:** 2026-09-18

**Authors:** Jonathan C. W. Liu, Ilona Gorbovskaya, Amy Y. Zhang, Margaret K. Hahn, Giada De Palma, Daniel J. Müller

**Affiliations:** 1 Department of Pharmacology and Toxicology, 7938 University of Toronto, Toronto, Canada; 2 Campbell Family Mental Health Research Institute, 7978 Centre for Addiction and Mental Health, Toronto, Canada; 3 Institute of Medical Sciences, 7938 University of Toronto, Toronto, Canada; 4 Department of Psychiatry, University of Toronto, Toronto, Canada; 5 Department of Medicine, 62703 Farncombe Family Digestive Health Research Institute, McMaster University, Hamilton, Canada; 6 Personalized Medicine, Ontario Shores Centre for Mental Health Sciences, Whitby, Canada

**Keywords:** schizophrenia, treatment-resistant, brain–gut axis, clozapine, gastrointestinal microbiome, microbiota, antipsychotic agents

## Abstract

**Background:**

Schizophrenia (SCZ) is a debilitating disorder affecting approximately 1% of the Canadian population and remains a major contributor to disease burden and disability. Up to one-third of individuals with SCZ are affected by treatment-resistant SCZ (TRS). Clozapine remains the most effective treatment for TRS. However, its use is limited by serious metabolic and immune-related side effects. The gut microbiome may be a potential contributor to both SCZ pathology and antipsychotic-induced side effects, though studies examining this in the clozapine-treated TRS population remain limited.

**Methods:**

This cross-sectional case-control study compared the gut microbiome composition of 25 TRS patients receiving long-term clozapine treatment to 25 healthy controls (HCs) matched for age, sex, body mass index, and smoking status. Participants provided fecal samples and completed assessments of psychiatric symptoms, eating behaviors, dietary intake, and metabolic measures. Fecal microbiome composition and predicted function were assessed using 16S rRNA gene sequencing (v3 region).

**Results:**

Individuals with TRS being treated with clozapine presented with a distinct microbiome in comparison to HC, including reduced microbial diversity. Taxonomic analyses revealed reduced relative abundance of Firmicutes, while elevated relative abundance of *Eggerthella* spp., *Mucispirillum* spp., and unclassified species within the order SHA-98 (class Clostridia). Exploratory analyses also identified nominal associations between select microbial features and clinical measures, including Positive and Negative Syndrome Scale (PANSS) scores and gastrointestinal symptoms. Predicted functional pathway analysis suggested alterations in microbial metabolic potential consistent with a metabolically stressed, pro-inflammatory microbiome.

**Conclusions:**

Individuals with clozapine-treated TRS demonstrate differences in gut microbiome composition compared to HC and are predicted to have some functional consequences relating to metabolic potential. These findings remain exploratory but contribute to a limited but growing body of literature in this clinically relevant population and provide a basis for longitudinal studies with appropriate comparator groups to better characterize these relationships and assess their clinical relevance.

## Plain Language Summary

Gut Bacteria Differences in People With Treatment-Resistant Schizophrenia Taking Clozapine

Schizophrenia is a serious mental health condition, and some individuals do not respond well to standard treatments. Clozapine is often used in these cases, but can cause side effects, including problems with metabolism and digestion. In this study, we examined the gut microbiome, a community of bacteria in the digestive system, in people with treatment-resistant schizophrenia who were taking clozapine, compared to healthy individuals. We found differences in the types and diversity of gut bacteria between the two groups. Some associations were also seen between gut bacteria and symptom measures, although these findings were exploratory. Because this study looked at data at a single point in time, we cannot determine cause and effect. Future studies following patients over time are needed to better understand how gut bacteria may relate to schizophrenia and clozapine effects.

## Résumé

**Contexte:**

: La schizophrénie est un trouble invalidant qui touche environ 1% de la population canadienne et qui contribue de façon importante au fardeau lié à la maladie et à l’invalidité. Jusqu’à un tiers des personnes atteintes de schizophrénie sont touchées par une schizophrénie résistante au traitement (SRT). La clozapine demeure le traitement le plus efficace contre la SRT. Cependant, son utilisation est limitée par de graves effets secondaires ne nature métabolique et immunitaire. Le microbiome intestinal pourrait jouer un rôle sur le plan de la pathologie de la schizophrénie et des effets secondaires causés par des antipsychotiques; toutefois, les études portant sur cette question dans la population de patients atteints de SRT récurrente traitée par la clozapine demeurent limitées.

**Méthodes:**

: Cette étude cas-témoins transversale visait à comparer la composition du microbiome intestinal de 25 patients atteints de SRT recevant un traitement à long terme par la clozapine à celle de 25 témoins sains appariés selon l’âge, le sexe, l’IMC et le tabagisme. Les participants ont fourni des échantillons de matières fécales et ont subi des évaluations des symptômes psychiatriques, des comportements alimentaires, de l’apport alimentaire et des fonctions métaboliques. La composition du microbiome fécal et son rôle prévu ont été évalués à l’aide du séquençage du gène de l’ARN recombinant 16S (région v3).

**Résultats:**

: Les personnes atteintes de SRT traitées par la clozapine présentent un microbiome différent de celui des témoins sains, y compris une réduction de la diversité microbienne. Les analyses taxonomiques ont révélé une diminution de l’abondance relative de *Firmicutes*, tandis qu’une abondance relative élevée d’*Eggerthella* spp. et de *Mucispirillum* spp. et d’espèces non classées appartenant à l’ordre SHA-98 (*Clostridia*). Les analyses exploratoires ont également permis d’établir des associations nominales entre certaines caractéristiques microbiennes et mesures cliniques, y compris les scores sur l’échelle des syndromes positifs et négatifs (PANSS) et les symptômes gastro-intestinaux (GI). L’analyse de la voie fonctionnelle prédite a révélé des altérations du potentiel métabolique microbien qui correspondraient à un microbiome pro-inflammatoire soumis à un stress métabolique.

**Conclusions:**

: La composition du microbiome intestinal des personnes atteintes d’une SRT traitée par la clozapine présente des différences par rapport au microbiome de témoins sains, ce qui est un facteur prédicteur de conséquences fonctionnelles liées au potentiel métabolique. Tout en demeurant exploratoires, ces résultats contribuent à un corpus limité, mais de plus en plus important, de documentation dans cette population pertinente sur le plan clinique et fournissent une base pour des études longitudinales auprès de groupes de comparaison appropriés permettant de mieux caractériser ces relations et d’évaluer leur pertinence clinique.

## Introduction

Schizophrenia (SCZ) is a chronic and severe psychiatric disorder with a prevalence of up to 1% of the population worldwide.^1^ SCZ encompasses a wide range of symptom domains and is generally treated with antipsychotic medications. While antipsychotic medication can be effective in reducing positive symptoms, its efficacy against negative symptoms and cognitive deficits is often limited and may not be effective in certain populations.^2–4^

Clozapine is widely considered the gold standard for treatment-resistant SCZ (TRS), demonstrating superior efficacy compared to other antipsychotic medications in reducing positive symptoms in addition to a lower risk of extrapyramidal symptoms. However, clozapine remains ineffective for many patients with TRS, with approximately 40% of patients failing to achieve an adequate or any response to treatment.^5,6^ Moreover, clozapine is associated with a unique adverse effect profile, including metabolic disturbances, weight gain, agranulocytosis, and cardiovascular events, which can limit its clinical utility.^7–9^ The ongoing need for tailored and adaptive treatment plans highlights the importance of understanding the underlying biological mechanisms of SCZ.

Recent research has begun to elucidate the intricate relationship between the gut microbiome and the brain, including its potential role in SCZ. The complex balance of the microorganisms residing in the gastrointestinal (GI) tract plays a crucial role in maintaining physiological homeostasis, influencing host metabolism and immune function, and even impacting brain function.^10–12^

A recent meta-analysis examining gut microbiota composition in SCZ suggests that SCZ patients exhibit a distinct gut microbiome composition compared to healthy controls (HCs), with antipsychotic medication having some influence on the shifts observed.^13^ The ten studies included compared patients with SCZ to HCs and found consistent shifts in microbial composition, with SCZ patients showing a reduction in microbes associated with anti-inflammatory and gut barrier-supporting function. However, the study did not find consistent differences in terms of overall richness and evenness of microbial species between the two groups. Some findings suggest that antipsychotic medications, including clozapine, may play a role in influencing gut microbiota composition.^13–16^ Overall, these findings suggest a complex interaction where SCZ patients may have a distinct gut microbiome composition and antipsychotics may induce microbial shifts, influencing therapeutic outcome and metabolic side effects.

This study aims to compare the gut microbiome composition of TRS patients on long-term clozapine treatment (minimum of six months) with matched HCs. We seek to identify specific microbial signatures associated with clozapine use and its commonly observed side effects. Based on previous literature, we expect to find altered gut microbial diversity and distinct community composition with a shift towards pro-inflammatory taxa in clozapine-treated TRS, consequently seeing distinct metagenomic profiles.^17–20^ Understanding microbiota changes associated with clozapine treatment and its side effects could offer novel insights into the biological pathways contributing to these side effects.

## Methods

### Study Design

This study is a cross-sectional case-control study conducted in the Centre for Addiction and Mental Health (CAMH) in Ontario, Canada. All subjects gave their informed consent to participate in this study. The study was approved by the CAMH Research Ethics Board (Ref#129/2015).

The two groups (clozapine-treated TRS patients and HCs) were compared through a single baseline visit, utilizing various clinical scales to measure eating behaviour, diet and nutrition, nicotine dependence, and psychiatric symptoms, along with assessments of anthropometric, metabolic, and gut microbial indices. All questionnaires were interview-administered.

Blood samples were collected and used for various measures whenever possible, including lipid profile, fasting blood glucose and insulin, hemoglobin A1C, and clozapine levels to provide insights into the metabolic health of the participants in relation to clozapine and the gut microbiome.

Recruitment for the study was done through various psychiatric outpatient clinics at CAMH and through the local community using posters and online advertisements. A total of 50 participants completed the study, 25 for each arm.

### Inclusion/Exclusion Criteria

Participants clinically diagnosed with SCZ, schizoaffective disorder, or psychosis not otherwise specified based on the *Diagnostic and Statistical Manual of Mental Disorders—Fifth Edition* (*DSM-V*), criteria were included in the clozapine arm. The inclusion criteria for this group were patients aged 18–45, taking clozapine monotherapy for a minimum of six months, and having the capacity to provide consent. Participants for the control arm were healthy individuals who did not have a current or history of psychiatric disorders based on *DSM-V* criteria. These individuals were matched to participants in the clozapine arm for age, sex, body mass index (BMI), and smoking status.

Participants were excluded from the study if they had a comorbid Axis I diagnosis, including a history of eating disorders; were using medication for metabolic disorders or infections; were taking food supplements; were pregnant or nursing; or had severe medical conditions or events that could significantly impact GI health.

### Questionnaires and Scales

The Dietary Questionnaire for Epidemiological Studies (DQES v2) was used to assess the dietary intake of participants over a specified period. Nutrient intake data such as macronutrients were provided by the Nutritional Assessment Office (Cancer Council, Victoria, Australia). The Dutch Eating Behavior Questionnaire (DEBQ) was used to evaluate the eating behaviours of participants.^21^ The Power of Food Scale (PFS) was used to assess the psychological influence of the food environment on participants’ eating behaviour.^22^

The Fagerström Test for Nicotine Dependence was used to evaluate the degree of nicotine dependence among the study participants, given that individuals with SCZ show higher rates of smoking and have been shown to affect microbiome composition.^23^

The Positive and Negative Syndrome Scale (PANSS) was administered to assess the severity of SCZ symptoms. The Subjective Well-Being Under Neuroleptic Treatment—short form (SWN-K) is a 20-item self-rating scale that assesses the patient's subjective effects of antipsychotics. The GI Rating Scale assessed the presence and severity of common GI symptoms, including abdominal discomfort, bloating, constipation, and diarrhea.^24^

### Microbial DNA and 16S rRNA and Statistical Analysis

Stool samples were obtained from participants within a 24-h window of the study visit, using the OMNIgene-GUT stool collection kit (OMR200, DNA Genotek). Samples were immediately stored at −80°C upon receipt until further processing. Total genomic DNA was extracted from the stool samples as previously described.^25^ Following this protocol, amplification of the V3 region of the 16S rRNA gene and Illumina sequencing were performed as previously described.^25,26^ Briefly, the data was analyzed following the pipelines of dada2 and QIIME2.^27,28^

Taxonomic assignments were performed using the RDP classifier with the Silva small subunit Ref. NR99 138.1 database (2020) training set.^29–31^ Analyses were done using either QIIME2, ANCOM plug-in for QIIME2, Phyloseq package (1.28) and MaAsLin2 for R (3.6.1), and SPSS software v. 23.^28,32,33^ Metagenomic functional content was predicted from 16S rRNA gene profile of samples using PICRUSt 2.0 (v. 2.4.1) and analyzed using MaAsLin2.^34,35^ Partial Spearman correlations, corrected by age and sex, were run between all microbial variables and clinical variables, such as BMI and PANSS, using the ppcor (1.1) package for R. All results were corrected for multiple comparisons. For MaAsLin2 comparisons, the q-value threshold for significance was set at 0.250, while for the rest of the comparisons, it was set at 0.05, allowing 5% of false discovery rate. The less stringent threshold for MaAsLin2 was selected to facilitate exploratory analysis of high-dimensional microbiome data while balancing type I and type II error in a relatively small sample, consistent with prior exploratory microbiome studies.^35^

Diversity of the gut microbiota can be measured through several metrics, including alpha diversity (within-sample), beta diversity (between-sample), and relative abundances of bacterial taxa. Alpha indices included the Shannon and Faith's index.^36,37^ Beta distance metric included Bray–Curtis dissimilarity.^38^

## Results

### Study Participants

A total of 50 participants were included in the study, comprising 25 individuals with TRS receiving long-term clozapine treatment and 25 HC (Table 1). The two groups were matched for age, sex, BMI, and smoking status. There were no significant differences between groups in age, sex distribution, BMI, or smoking prevalence. However, a greater proportion of clozapine-treated TRS patients self-identified as being of European ancestry.

**Table 1.** Demographic and Clinical Characteristics of Participants.

Variable	Schizophrenia Patients on Clozapine (*n* = 25) Mean (SD)	Matched Healthy Controls (*n* = 25) Mean (SD)s	*P*-Value
Age (years)	33.6 (6.4)	33.1 (6.0)	0.786
Sex (% male)	17 (68%)	17 (68%)	-
Ethnicity (% European)	14 (56%)	8 (32%)	0.154
Height (cm)	173.2 (10.4)	169.5 (9.8)	0.202
Weight (kg)	87.0 (18.6)	81.6 (16.8)	0.288
BMI	29.0 (5.5)	28.4 (5.3)	0.676
Waist to hip ratio	0.95 (0.07)	0.95 (0.08)	0.747
Smoking status (% smokers)	7 (28%)	7 (28%)	-
Clozapine dose (mg)	277.9 (140.9)	-	-
Clozapine plasma levels (nmol/L)	1388.9 (677.7)	-	-

TRS patients on clozapine treatment exhibited relatively stable symptomatology (Table 2), as reflected in mild PANSS scores and subjective well-being (SWN-K). Despite this clinical stability, they reported more GI symptoms (W = 126, *P* = 0.0002; Wilcoxon rank-sum test) and were less likely to engage in regular exercise when compared to the control group. Clozapine-treated TRS patients also demonstrated distinct eating behaviors, with a tendency toward greater food-related impulsivity and higher emotional or external eating behaviors, as indicated by elevated PFS and DEBQ scores. However, these differences were not statistically significant.

**Table 2.** Summarized Clinical and Behavioral Questionnaire Scores of Participants.

Clinical Measure	Schizophrenia Patients on Clozapine (*n* = 25) Mean (SD)	Matched Healthy Controls (*n* = 25) Mean (SD)	*P*-Value
Fagerström Nicotine Dependence Scale	3.3 (1.9)	2.2 (1.7)	0.675
Exercise/Physical Activity Evaluation (% active)	10 (40%)	14 (56%)	0.396
Dutch Eating Behavior Questionnaire	75.0 (21.2)	66.1 (21.1)	0.162
Power of Food Scale	50.9 (15.4)	45.0 (13.6)	0.157
Gastrointestinal Symptom Rating Scale	24.5* (**9.1)**	17.1* (4.8)	**< 0.001**
Subjective Well-Being under Neuroleptic Treatment Scale	92.9 (14.1)	-	-
PANSS: positive	10.5 (3.6)	-	-
PANSS: negative	14.6 (5.7)	-	-
PANSS: general	24.5 (5.6)	-	-
PANSS: total	49.6 (11.2)	-	-

PANSS = Positive and Negative Syndrome Scale.

### Clozapine Exposure Is Associated With GI Symptom Severity

To further examine relationships between GI symptoms and clozapine exposure, we performed both Pearson and Spearman correlations between GI symptom scores and clozapine-related predictors (daily dose, plasma clozapine and norclozapine levels, dose-to-metabolite ratio, and duration of clozapine treatment).

In the full sample (clozapine-treated TRS and HC combined), GI symptom severity showed significant positive correlations with all predictors using Spearman correlation. When restricting the analysis to clozapine-treated TRS patients only, associations were attenuated after FDR correction. Clozapine dose showed a modest inverse association with GI symptoms (*r* = −0.38, *P* = 0.060), and plasma levels of clozapine and norclozapine also showed a trend toward a negative association. Time on clozapine remained positively associated with GI symptoms, but did not reach significance (*r* = 0.40, *P* = 0.14).

### The Gut Microbiome of Patients With Clozapine-Treated TRS Differs From That of Healthy Controls

Twenty-five clozapine-treated TRS patients and 25 HC fecal samples were analyzed using 16S rRNA sequencing on the Illumina platform. After quality filtering, a total of 3,273,786 reads were retained, with an average of 65,475.72 reads per sample. From these, we identified 1,644 amplicon sequence variants (ASVs), with an average of 136.54 ASVs per sample. The initial ASV table was normalized using two approaches: rarefaction to a single even sequencing depth and calculating relative abundance of each ASV. Both normalization methods produced comparable results.

**Figure 1.**
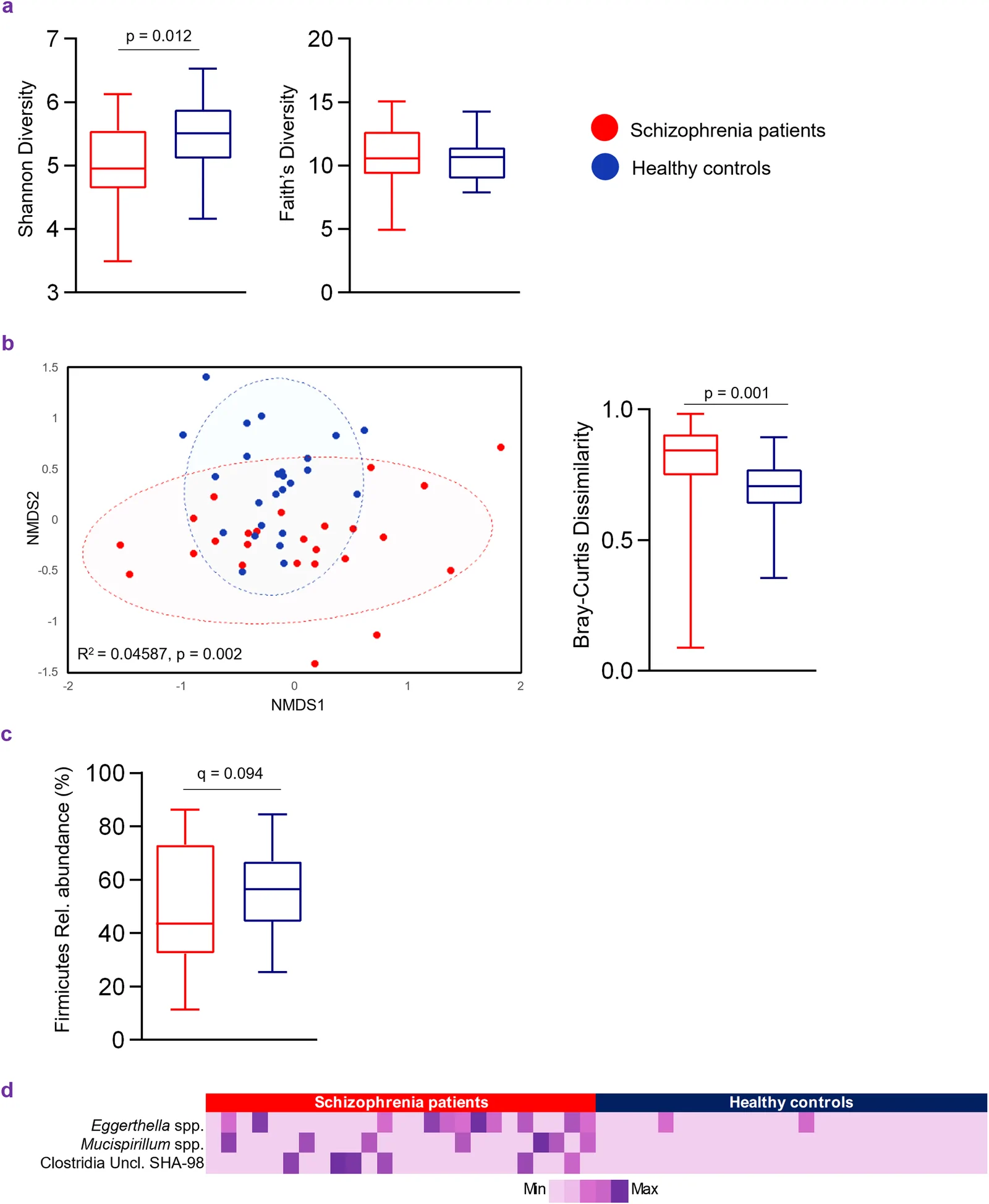
(a) α-diversity comparisons between SCZ patients and HCs. α diversity of the gut microbiota in SCZ patients (red) and HCs (blue) is represented through two metrics: Shannon diversity index (left) and Faith's phylogenetic diversity index (right). SCZ patients exhibited significantly lower Shannon diversity (*P* = 0.012). (b) β-diversity comparison between SCZ patients and HCs. Non-Metric Multidimensional Scaling (NMDS) plot (left) illustrates distinct microbial community structures (R^2^ = 0.04587, *P* = 0.002) based on Bray–Curtis dissimilarity, with increased intra-group variation among TRS patients (right, *P* = 0.001). (c) Relative abundance of the phylum Firmicutes was lower in SCZ patients compared to HCs (Q = 0.094). (d) Heatmap showing higher relative abundance of Eggerthella spp., Mucispirillum spp., and unclassified Clostridia (SHA-98) in SCZ patients compared to HCs.

We found that clozapine-treated TRS patients had a significantly lower microbial diversity compared to HCs, as assessed by the Shannon alpha diversity Index (*P* = 0.012, Figure 1a). However, no significant difference was observed when assessing microbial diversity with Faith's phylogenetic diversity index between the two groups (Figure 1a).

SCZ = schizophrenia; HCs = healthy controls; TRS = treatment-resistant schizophrenia.

The microbiome of patients with clozapine-treated TRS was different from that of HCs, as shown by the two separate clusters in the beta diversity plots constructed on the Bray–Curtis dissimilarity matrix (R^2^ = 0.046, *P* = 0.002; Figure 1b). Notably, SCZ patients exhibited significantly greater intra-group variation compared to controls (Figure 1b).

Analysis of the microbiome composition of HCs and patients with SCZ, identified three potential genera of interest (Figure 1d). We found that patients with clozapine-treated TRS had a significant decrease in relative abundance of bacteria belonging to the phylum Firmicutes (Figure 1c), while presenting with a significant increase in the relative abundance of *Eggerthella* spp., *Mucispirillum* spp., and unclassified species from the order SHA-98 of the class Clostridia (Figure 1d).

**Figure 2.**
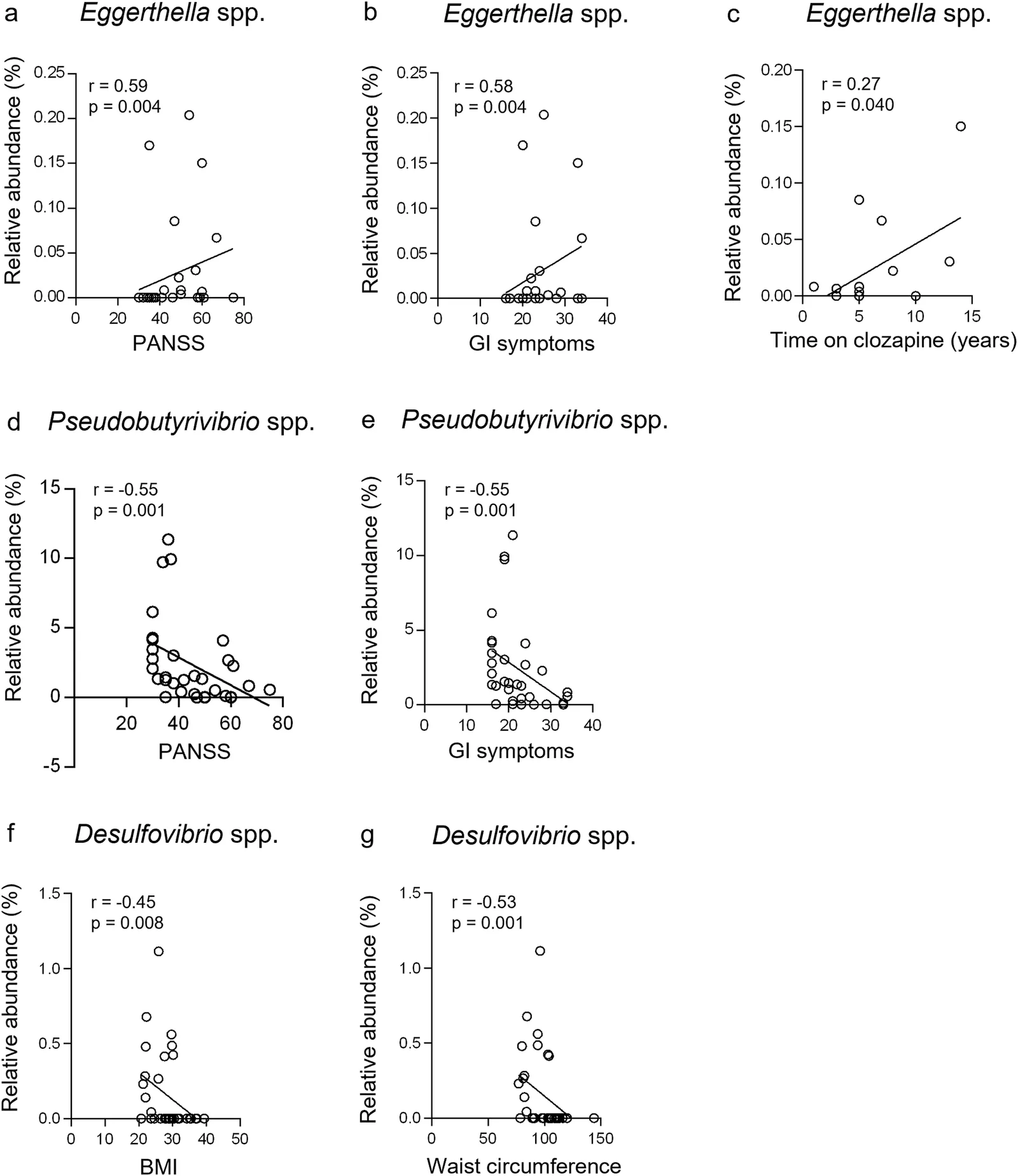
Correlations between microbial taxa and clinical or metabolic measures in SCZ patients. (a–c): Eggerthella spp. relative abundance was positively correlated with PANSS total score (*r* = 0.59, *P* = 0.0035), GI symptom severity (*r* = 0.58, *P* = 0.004), and duration of clozapine treatment in years (*r* = 0.27, *P* = 0.04). (d–e) Pseudobutyrivibrio spp. relative abundance was negatively correlated with PANSS scores (*r* = –0.55, *P* = .0012) and GI symptom severity (*r* = –0.55, *P* = .001). (f–g) Desulfovibrio spp. abundance was negatively correlated with BMI (*r* = –0.45, *P* = .008) and waist circumference (*r* = –0.53, *P* = .001). These associations were nominally significant and did not survive correction for multiple comparisons.

We performed correlation analyses to explore the relationships between bacterial abundances and various clinical, dietary, and metabolic measures in both groups. *Eggerthella* relative abundance was positively correlated with the severity of PANSS scores (*r* = 0.59, *P* = 0.0035, adjusted for sex), with GI symptom severity (*r* = 0.58, *P* = 0.004, adjusted for sex), and with duration of clozapine treatment measures in years (*r* = 0.27, *P* = 0.04) (Figure 2a). Conversely, *Pseudobutyrivibrio* relative abundance was negatively correlated with PANSS scores (*r* = −0.55, *P* = 0.0012), and GI symptom severity (*r* = −0.55, *P* = 0.001) (Figure 2b). Additionally, we observed significant negative correlations between *Desulfovibrio spp.* and metabolic measures, BMI (*r* = −0.45, *P* = 0.008) and waist circumference (*r* = −0.53, *P* = 0.001) (Figure 2c). However, these results did not survive correction for multiple comparisons.

PANSS = Positive and Negative Syndrome Scale; SCZ = schizophrenia; HCs = healthy controls; TRS = treatment-resistant schizophrenia; GI = gastrointestinal.

### Inferred Metagenomic Composition

**Figure 3.**
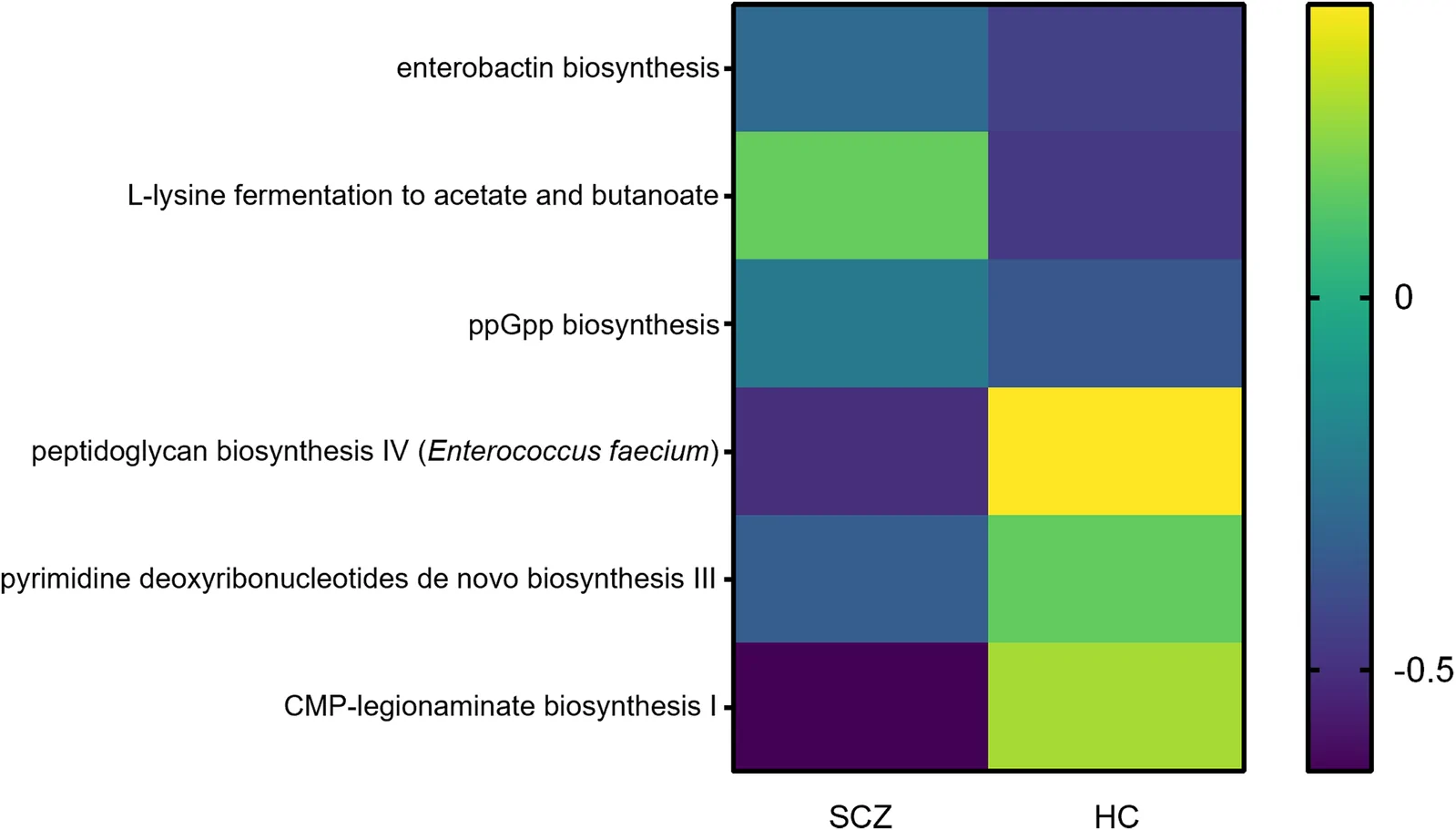
Differential abundance of microbial metabolic pathways in SCZ patients and HCs. Heatmap depicting the relative abundance of selected microbial pathways inferred from 16S rRNA gene sequencing data in SCZ patients and HCs. Colour scale reflects normalized relative abundance differences across groups.

Beyond taxonomic differences, clozapine-treated TRS patients were predicted to exhibit altered microbial functional pathways, as determined through analysis of PICRUSt2-inferred metabolic pathways. Six predicted pathways differed between clozapine-treated TRS patients and matched HCs (Figure 3). Clozapine-treated TRS patients exhibited reduced predicted abundance of pathways involved in peptidoglycan biosynthesis IV, pyrimidine deoxyribonucleotide de novo biosynthesis, and CMP-legionaminate biosynthesis I. In contrast, predicted pathways related to enterobactin biosynthesis, L-lysine fermentation to acetate and butanoate, and ppGpp biosynthesis were relatively enriched in this cohort compared to controls. These findings reflect predicted functional potential rather than directly measured metabolic activity. However, they suggest that GMB composition differences may result in alterations in inferred metabolic capacity, the biological implications of these findings remain uncertain and warrant further investigation through direct approaches.

## Discussion

### Microbial Diversity (α-Diversity) and Community Structure (β-Diversity) in Clozapine-Treated TRS

In this study, we investigated potential microbiome alterations in clozapine-treated TRS patients compared to HC, with the goal of gaining insight into the broader metabolic and physiological changes typically observed in this group. As all patients were receiving long term-clozapine, these findings cannot be attributed specifically to SCZ, treatment resistance, or medication effects in isolation, but likely reflect the combined and interacting influence of these factors.

Our findings revealed microbiome alterations in TRS patients receiving chronic clozapine treatment. We saw evidence for a reduction in microbial richness and evenness in clozapine-treated TRS patients compared to HCs, which is consistent with findings in the SCZ population, though it contrasts with two studies specifically looking at the TRS population being treated with clozapine where no difference was reported.^13,16,39^ These discrepancies may reflect differences in study design, participant characteristics, and potentially duration of clozapine treatment. We found a distinct microbiome shift in the patients being treated with clozapine who also exhibited a more variable community compared to HCs, consistent with various studies looking at the microbiome in SCZ patients.^13,39^ To better understand the nature of the observed shifts, we examined taxonomic profiles and relative abundances to identify specific bacterial groups associated with clozapine-treated TRS.

### Microbial Signatures Associated With Clozapine-Treated TRS and Symptom Severity

Clozapine-treated TRS patients showed increased relative abundance of *Eggerthella* spp., aligning with prior reports of *Eggerthella* elevation in SCZ and other psychiatric disorders.^17,18^ *Eggerthella* is associated with intestinal inflammation, immune modulation, and mucosal barrier disruption, frequently reported to be elevated in SCZ spectrum disorders and inflammatory bowel disease.^40,41^ In our samples, *Eggerthella* relative abundance was also positively associated with greater psychiatric and GI symptom severity.

Conversely, we saw a negative association between psychiatric and GI symptom severity with relative *Pseudobutyrivibrio* spp. abundance, a producer of short-chain fatty acids, specifically butyrate.^42,43^ Previous studies have reported reduced levels of butyrate-producing bacteria similar to *Pseudobutyrivibrio* in SCZ.^19,20,44^ Reduced levels of butyrate have been associated with increased gut permeability and systemic inflammation, processes that have been implicated in SCZ.^45,46^ These findings remain nominal and cross-sectional, not allowing inference of causality or directionality. However, it still highlights possible microbial patterns in this underrepresented patient group that may merit further investigation to distinguish underlying biology, medication exposure, or downstream effects of the chronic disorder and clinical management.

### Predicted Bacterial Functional Shifts and Potential Host Implications in Clozapine-Treated TRS

Beyond taxonomic shifts, we identified differences in predicted microbial metabolic pathways between clozapine-treated TRS patients and HCs. These findings are based on PICRUSt2-inferred pathways derived from 16S rRNA gene data and therefore reflect predicted microbial functional potential rather than directly measured metabolic activity.

We saw a reduction in predicted pathways related to peptidoglycan biosynthesis IV and pyrimidine deoxyribonucleotide de novo biosynthesis III in clozapine-treated TRS patients. Peptidoglycan biosynthesis is essential for cell wall integrity and structural stability in bacteria, while de novo synthesis of nucleotides is an energy-intensive process.^47–50^ Elevated oxidative stress and chronic inflammation have been reported in SCZ, which may be associated with altered microbial environments.^51–53^ Whether the predicted pathway differences observed in the present study relate to these processes remains uncertain.

In contrast, we saw an elevation in predicted pathways related to ppGpp biosynthesis, enterobactin biosynthesis, and L-lysine fermentation, which play roles in bacterial survival under stress conditions, inflammatory conditions, and short-chain fatty acid production, respectively.^48,54–58^ These observed GMB shifts may reflect differences in predicted microbial functional potential. However, their relevance to host-level processes remains uncertain and cannot be used to infer direct biological effects without validation through metabolomic or other functional approaches.

### Exploring Clozapine-Related Contributors to GI Disturbances

In addition to microbiome alterations, we explored whether clinical indicators of clozapine exposure were associated with GI symptom severity. In the full cohort, GI symptom scores were positively correlated with clozapine treatment duration, daily dose, and plasma clozapine and norclozapine levels. As clozapine exposure variables are not applicable to controls, despite measured values of zero, these associations reflect group-level differences rather than a dose–response relationship. When restricting the analysis to the clozapine-treated TRS subgroup, many of these associations were attenuated, suggesting that GI symptoms may reflect broader disorder-, treatment-, or lifestyle- related factors that differ between groups, rather than dose-dependent effects. This may reflect clinical factors such as dose adjustment in response to side effects or tolerability, survivorship bias among individuals maintained on long-term treatment, or symptom adaptation. These observations align with existing reports of clozapine-associated GI dysmotility and suggest that GI symptom severity may not increase proportionally with dose or plasma concentration once treatment is established.^59,60^ Longitudinal studies are required to better characterize how GI symptoms relate to clozapine exposure and disorder-related characteristics over time.

### Clinical Implications, Strengths, and Limitations

To date, only a few studies have investigated gut microbiota in relation to TRS and long-term clozapine use (O’Donnell et al. 2022; Vasileva et al. 2024; Yin et al. 2023).^15,16,39^ Our findings contribute to the growing evidence suggesting that microbiome differences are observed in individuals with clozapine-treated TRS, likely reflecting the combined influence of chronic disorder, medication exposure, and associated lifestyle and metabolic factors.

A major strength of this study is its focus on a unique and clinically homogeneous population of TRS patients receiving clozapine treatment. Despite their unique clinical profile and high burden of metabolic comorbidities, these individuals remain underrepresented in microbiome research. Another strength of this study is the extensive clinical characterization of participants, including a broad set of validated psychiatric, GI, and lifestyle-related questionnaires. This allowed for exploratory associations between microbiota and clinically meaningful symptom domains.

A key limitation of this study is its cross-sectional design, which limits observing microbiome changes over time and determining if observed differences preceded or result from clinical and treatment-related factors. As all patients were receiving long-term clozapine and no comparator group of non-clozapine-treated SCZ patients was included, we cannot distinguish the independent effects of SCZ or treatment-related factors on the GMB. Accordingly, the present study should be interpreted as characterizing the gut microbiome profile of individuals with chronic clozapine-treated TRS, rather than identifying microbiome changes that are specific to clozapine treatment. The possibility of false positive findings remains, particularly given the number of analyses conducted in a relatively small sample and should be interpreted with caution. Functional inferences should be interpreted as indirect and exploratory since 16S rRNA gene sequencing provides limited taxonomic and functional resolution and cannot directly assess microbial metabolic activity. Differences in ethnicity between groups, including a higher proportion of European ancestry in the clozapine-treated TRS group, may represent a potential confounding factor but were not included as a covariate in the primary analyses due to the modest sample size. Although dietary intake and exercise habits were assessed, these variables were not included in the adjusted analyses. Given their established influence on gut microbiome composition, their potential confounding effects cannot be excluded.

## Conclusions

In summary, our study identifies GMB differences in individuals with TRS receiving chronic clozapine treatment compared to HCs. These findings are exploratory and should be interpreted cautiously, as the current study design does not allow for distinguishing disorder-related effects from those associated with medication exposure. Nonetheless, these results contribute to a limited body of literature in this clinically relevant population and highlight the need for larger, well-powered cohort studies incorporating longitudinal designs and an appropriate non-clozapine comparator group to better characterize, validate, and differentiate between these effects.
